# Kinase Activity of ArcB from *Escherichia coli* Is Subject to Regulation by Both Ubiquinone and Demethylmenaquinone

**DOI:** 10.1371/journal.pone.0075412

**Published:** 2013-10-07

**Authors:** Poonam Sharma, Stefan Stagge, Martijn Bekker, Katja Bettenbrock, Klaas J. Hellingwerf

**Affiliations:** 1 Molecular Microbial Physiology Group, Swammerdam Institute for Life Sciences, University of Amsterdam, and Netherlands Institute for Systems Biology, Amsterdam, the Netherlands; 2 MPI für Dynamik Komplexer Technischer Systeme, Experimentelle Systembiologie, Magdeburg, Germany; University of Strathclyde, United Kingdom

## Abstract

Expression of the catabolic network in *Escherichia coli* is predominantly regulated, via oxygen availability, by the two-component system ArcBA. It has been shown that the kinase activity of ArcB is controlled by the redox state of two critical pairs of cysteines in dimers of the ArcB sensory kinase. Among the cellular components that control the redox state of these cysteines of ArcB are the quinones from the cytoplasmic membrane of the cell, which function in ‘respiratory’ electron transfer. This study is an effort to understand how the redox state of the quinone pool(s) is sensed by the cell via the ArcB kinase. We report the relationship between growth, quinone content, ubiquinone redox state, the level of ArcA phosphorylation, and the level of ArcA-dependent gene expression, in a number of mutants of *E. coli* with specific alterations in their set of quinones, under a range of physiological conditions. Our results provide experimental evidence for a previously formulated hypothesis that not only ubiquinone, but also demethylmenaquinone, can inactivate kinase activity of ArcB. Also, in a mutant strain that only contains demethylmenaquinone, the extent of ArcA phosphorylation can be modulated by the oxygen supply rate, which shows that demethylmenaquinone can also inactivate ArcB in its oxidized form. Furthermore, in batch cultures of a strain that contains ubiquinone as its only quinone species, we observed that the ArcA phosphorylation level closely followed the redox state of the ubiquinone/ubiquinol pool, much more strictly than it does in the wild type strain. Therefore, at low rates of oxygen supply in the wild type strain, the activity of ArcB may be inhibited by demethylmenaquinone, in spite of the fact that the ubiquinones are present in the ubiquinol form.

## Introduction

The enterobacterium *Escherichia coli* is a widely studied model organism, not only because of fundamental interests, but also due to increasing societal demand for a number of (relatively reduced) products of its catabolism, such as organic acids and alcohols which can be obtained in high yield through metabolic engineering. *E. coli* can operate in three basic modes of metabolism: aerobic respiration, anaerobic respiration and fermentation. Depending on the availability of oxygen, or of alternative electron acceptors like nitrate and fumarate, *E. coli* switches between these modes and therefore requires a well-orchestrated gene expression repertoire to accomplish this switch [1]. The major differences between these modes of metabolism are in the pathway(s) used for production of ATP and generation of reducing power in the form of NAD(P)H. While oxygen is *E. coli*’s preferred final electron acceptor, in its absence, alternative electron acceptors such as fumarate, nitrate, DMSO and TMAO can be used instead [2]. During growth, catabolic activity in the cytoplasm liberates electrons in the form of reduced electron carriers such as NADH and FADH_2_, which transfer the electrons to the quinone pool(s) in the respiratory chain via specific dehydrogenases. Quinols then transfer the electrons to the final electron acceptor oxygen, or to alternate acceptors, via quinol oxidase complexes or dedicated reductases [3]–[5].

Quinones are lipid-soluble electron, or better said ‘hydrogen’ carriers that reside in the cytoplasmic membrane and function as mobile redox carriers in the respiratory chain. *E. coli* contains three different types of quinone: ubiquinone (UQ), demethylmenaquinone (DMK) and menaquinone (MK), where DMK is the biosynthetic precursor of MK. The redox midpoint potential of these three quinones is 110 mV, 40 mV and −80 mV, respectively [2].

Depending on the midpoint potential of a specific dehydrogenase/oxidase combination, we expect that the quinone that will preferably function as their mobile electron carrier, will differ [6], [7]. This is consistent with the observation that DMK and MK are abundant under anaerobic conditions when the redox potential of the electron acceptor may be more negative than the one of ubiquinone. Conversely, ubiquinone is the most abundant redox carrier during aerobic growth which is consistent with the high redox mid-point potential of the oxygen/water couple [7].

The ArcB/ArcA two-component signal transduction system (anoxic redox control) is an indirect oxygen sensor and functions as a transcriptional regulator of the oxidative- and fermentative catabolism in *E. coli* [8]–[10]. It consists of a trans-membrane sensor, ArcB, and a transcriptionally active response regulator, ArcA. ArcB is present in the cytoplasmic membrane in homo-dimeric form and contains cysteines/disulphide bonds that can be oxidized/reduced by quinones/quinols. Under oxidizing conditions, the kinase activity of ArcB is inhibited by the oxidation of two cysteine residues that each can form an intermolecular disulphide bond upon oxidation, which then leads to de-phosphorylation of ArcA-P via an Asp^54^ His^717^ Asp^576^ Pi phosphorelay [11]–[13]. In its active state, elicited for example by oxygen limitation, auto-phosphorylation of ArcB takes place at the expense of ATP. This in turn leads to trans-phosphorylation of ArcA via the transfer of the phosphoryl-group from His^717^ of ArcB to the aspartate residue (Asp^54^) in the active site of ArcA [12], [14], [15]. In its phosphorylated state ArcA has an increased affinity for its DNA targets and acts both as a positive and as a negative transcriptional regulator for genes that are involved in a wide variety of metabolic pathways [9], [16], [17]. An additional form of regulation of ArcB may be exerted by a number of metabolites (e.g. D-lactate, acetate and pyruvate) that play a role in fermentation [18]–[20].

Georgellis and coworkers observed in *in vitro* experiments that oxidized ubiquinone (250 µM of UQ_0_) inhibits the kinase activity of ArcB, which led them to the prediction that ArcB activation will decrease in parallel with the lowering of the reduction level of the ubiquinone pool [21], [22]. However, Alexeeva *et al*., in a detailed reporter-enzyme study of the Arc system in which the rate of oxygen supply to chemostat-grown cells was dosed with high precision, showed that the level of Arc activation is highest under specific micro-aerobic conditions with a second, but lower, maximum under anaerobic conditions [23]. In a follow-up of this work, Bekker *et al*. speculated that not only ubiquinone but also oxidized menaquinone may de-activate ArcB, which then might explain the decrease in ArcB activity at low oxygen-supply rates [24].

Here we report on further experiments aimed at resolving the role of the three types of quinone in the regulation of ArcB in *E. coli*. To this end our wild type *E. coli* (MG1655), which contains all three types of quinone, has been compared with mutants containing UQ only, DMK only, or a combination of DMK and MK only (note that because DMK is the biosynthetic precursor of MK it is nearly impossible to construct mutants containing exclusively MK [7]). These different strains of *E. coli* were compared with respect to growth rate, quinone content, quinone redox state, ArcA-phosphorylation level, and ArcA-dependent gene expression. The results obtained unequivocally show the ability of UQH_2_ to activate, and DMK to inactivate ArcB, and thereby provide experimental evidence that supports the model proposed by Alexeeva *et al.* [23].

## Materials and Methods

### Strains Used

The *E. coli* K12 strain MG1655, containing all three types of quinone, was used as the wild type strain. Deletion mutants containing defects in biosynthesis of UQ, MK, and DMK plus MK were constructed by P1 phage transduction of the desired mutations (Table 1). A *ubiCA* deletion strain received from Dr. Robert Poole [25] was used to transduce MG1655, to make AV33. Strains from the KEIO collection with a *menA-* or a *ubiE* deletion were used to construct strains AV34 and AV36, respectively. Mutants were checked by PCR, followed by phenotypic analysis of the quinone pool(s) present in the respective strains, using HPLC (as described below under *Quinone extraction and analysis*).

**Table 1:** List of the strains used in this study.

Strain	Genotype	Quinones present
**MG1655**	K-12 wild type	UQ+DMK+MK
**AV33**	MG1655, *ΔubiCA::kan*	DMK+MK
**AV34**	MG1655, *ΔmenA::kan*	UQ
**AV36**	MG1655, *ΔubiE::kan*	DMK

### Batch Culture

Cells were grown in batch culture at 37°C using Evans salt medium with nitrilo-acetic acid (2 mM) and sodium phosphate buffer (100 mM, pH 7) to increase buffering capacity [26]. Glucose (20 mM or 50 mM) was used as carbon source and LB (1% (v/v)) was occasionally added to enhance the growth rate of the cells, which was particularly relevant for the three quinone deletion strains. During aerobic growth, aeration was accomplished by growing 100 ml culture volumes in 1000 ml Erlenmeyer flasks in a rotary shaker at 200 rpm. Cultures were inoculated from LB plates. For anaerobic growth, 50 ml cultures were grown overnight in 50 ml Greiner tubes in Evan’s medium supplemented with 50 mM glucose and 1% (v/v) LB, the contents of which were then transferred to a batch fermenter containing 500 ml of Evan’s medium with the same medium and continuous nitrogen gas sparging at a flow rate of 50–80 ml/min to maintain anaerobic conditions. In anaerobic cultures containing fumarate as the electron acceptor, fumarate was added in the concentration of 50 mM to the medium, keeping all the other conditions the same.

### Quinone Extraction and Analysis

The extraction and analysis of quinones was accomplished essentially as described in Sharma et al. 2012 [7]. Briefly, at each time point a 2 ml sample was taken in 6 ml of a 1∶1 (v/v) mixture of ice-cold methanol (for quenching) and petroleum ether (for dissolving the quinones and other hydrophobic compounds). The mixture then was vortexed for 1 minute and centrifuged at 3,000 rpm for 1 minute. Then the upper petroleum ether phase was transferred with a Pasteur pipet, under a nitrogen atmosphere, into a glass tube. The procedure was repeated once to ensure complete extraction of the quinones. The samples were dried under nitrogen gas and the extracts were then stored at −20°C until analysis.

Before fractionating the samples with high-performance liquid chromatography (HPLC; Pharmacia LKB 2249 gradient pump system with an LKB 2151 variable-wavelength monitor) using a reversed-phase Lichrosorb (Chrompack, Bergen op Zoom, The Netherlands) RP10 C18 column (size, 4.6 mm; interna1 diameter, 250 mm), the extracted quinones were re-suspended using a glass rod, in 80 µl ethanol. The column was equilibrated with pure methanol as the mobile phase at the flow rate of 2 ml/min. Detection of the quinones was performed at 290 nm for ubiquinone (UQ) and at 248 nm for napthaquinones (DMK and MK). The amount of each quinone species was calculated from the relevant area under the peak. Peaks were identified by UV/visible spectroscopy and tandem mass spectral analysis. The peaks around 8.6 and 13.7 minutes elution time showed spectra for UQ_8_H_2_ and UQ_8_, respectively, whereas peaks eluting at around 20.9 and 24.4 minutes revealed spectra of DMK_8_ and MK_8_, respectively. In the case of UQ_8_, both the reduced- and the oxidized form can be detected. For DMK_8_ and MK_8_ however, only the amount of the oxidized form can be quantified reliably, probably due to rapid auto-oxidation of the napthaquinones. The amount of quinones was calculated from the area under the peak by using UQ_10_ and MK_4_ as calibration standards.

### Measurement of ArcA Phosphorylation

ArcA phosphorylation levels were measured with Phos-tag™-acrylamide gel electrophoresis and Western immunoblotting as described by Rolfe *et al.* [1]. For sample collection, every hour a 5 ml sample from the culture was directly quenched in 1 ml formic acid (6 M) plus 100 µl chloramphenicol solution (25 mg/ml), to make a final concentration of ∼1 M formic acid. The latter acts to stabilize phospho-Asp residues, whereas chloramphenicol prevents further protein synthesis. The samples were then centrifuged at 4,000 rpm for 5 minutes and the supernatant was removed. Pellets were re-suspended in 50 µl 1 M formic acid, and stored at −80°C until use for protein sample processing.

Protein sample processing for the detection of the mobility shift of phosphorylated ArcA was carried out with polyacrylamide-bound Mn^2+^-Phos-tag SDS gels. For gel preparation a 10% (w/v) solution of acrylamide/bis-acrylamide was used for the resolving gel, to which 5 mM Phos-tag-acrylamide (final concentration 25 µM) and MnCl_2_ (50 µM final concentration) was added. In the gel-electrophoresis device, the resolving gel was cast in the chamber until 75% of the gel chamber size. For the upper part of the chamber a stacking gel of 3% (w/v) acrylamide was used. The gel was placed in the running device with SDS running buffer [27].

Before loading the gel, samples were diluted to an optical density of approximately 4, at 600 nm, in a v/v mixture of 61.5% 1 M formic acid, 33% loading buffer and 5.5% 10 M NaOH, as described by Barbieri *et al.* [27]. Of each sample 5 µl was loaded per well, and 4 µl stained PAGE markers were run separately. The gel was run at 12 mA until the loading dye was 0.5 cm above the bottom of the gel. Gels were then washed in transfer buffer containing 1 mM EDTA for 15 minutes and subsequently washed in transfer buffer only. The gel was blotted onto a nitrocellulose membrane, using wet blotting, with a transfer buffer containing methanol (20% (v/v)), SDS (0.04% (w/v)), tris (0.3% (w/v)) and glycine (1.5% (w/v)), overnight at 20 V.

After blotting, the membrane was blocked with milk powder to prepare it for Western analysis. The membrane was first hybridized with a specific rabbit anti-ArcA antibody at a dilution of 1∶10,000. Secondly, a 1∶5,000 dilution of secondary antibody, horseradish peroxidase-conjugated goat anti-rabbit IgG (Bio-Rad), was added and visualized with a chemi-luminescence assay based on peroxidase activity. In this gel system the phosphorylated- and non-phosphorylated form of ArcA run at an apparent size of approximately 34 and 28 kDa, respectively. The relative level of ArcA phosphorylation was calculated by analysis of the relevant band intensities using a Phosphor-Imager and software for the analysis of Western blots from Image studio (Table S1).

## Results and Discussion

### Physiological Characterization of Wild Type- and Mutant Strains

In order to get more insight into the regulation of ArcB activity through the relationship between ArcA phosphorylation and the redox state of the three different quinone pools, three different mutant strains of *E. coli,* and the corresponding wild type strain, were physiologically characterized with respect to growth rate via optical density measurements at 600 nm. Table 2 shows the specific growth rate of the four strains under the conditions tested. The results clearly show that the growth rate attained under aerobic conditions in MG1655 (wild type), and in the three quinone mutants, is much higher than under anaerobic conditions, irrespective of the quinone species present. The growth rate of AV36 (*ΔubiE*) and AV34 (*ΔmenA*) is similar under aerobic conditions, again showing the functionality of DMK in aerobic respiration [7]. As expected, growth with anaerobic respiration is more efficient than with fermentation. Interestingly, differences between the strains’ growth rates are small, confirming [28] that many permutations and combinations are possible for transfer of electrons from dehydrogenases - via (a) quinone(s) - to a terminal acceptor.

**Table 2:** Physiological analysis of the strains used in this study.

Strain	aerobic	anaerobic +fumarate	Anaerobic
**Wild type**	0.65±0.01	0.21±0.02	0.15±0.02
***ΔubiCA***	0.43±0.03	0.22±0.06	0.20±0.02
***ΔmenA***	0.49±0.01	0.17±0.04	0.13±0.02
***ΔubiE***	0.49±0.05	0.20±0.06	0.13±0.05

Growth rates (hr^−1^) for MG1655 (Wild type) and its quinone deletion mutants during exponential growth in Evan’s medium supplied with 50 mM glucose and 1% LB at 37°C under aerobic, anaerobic and anaerobic plus 50 mM fumarate conditions. The values represent the mean of measured values from biological triplicates with standard deviation.

### Quinone Content of the Cells Grown under Various Conditions

Upon changes in availability of the final electron acceptor, the composition of the quinone pools was shown to adjust rapidly, presumably due to regulation at the post-translational level [29], [30]. In agreement with previous results [30] Table 3 shows that in the wild type organism, MG1655, ubiquinones are most abundant under aerobic conditions, and the menaquinones are most abundant under anaerobic conditions. Table 3 also shows that in the presence of fumarate as specific electron acceptor, the cellular content of DMK and MK decreases. The molecular basis for these differences in quinone production level is still largely unknown. Nevertheless, it is important to measure them for a detailed interpretation of the mechanism of regulation of ArcB activity. In the relevant strains under aerobic conditions ubiquinone is most abundant (i.e. WT and *ΔmenA)*, while anaerobically menaquinones are most abundant (in WT, *ΔubiE and ΔubiCA)* (Table 3). Both DMK and MK are present in significant amounts under all relevant conditions tested (i.e. in WT, *ΔubiE and ΔubiCA*), indicating that menaquinone is particularly abundant anaerobically. These results are in agreement with the results of [24] who reported an increase in MK towards anaerobiosis. However, the small decrease in DMK content that they observed is not observed in this experiment. Significantly, the level of napthaquinones (i.e. DMK plus MK) is significantly up-regulated upon impairment of ubiquinone synthesis (compare WT with *ΔubiCA*) both aerobically and anaerobically, but not when fumarate is added as an external electron acceptor (Table 3).

**Table 3:** Relationship between quinone concentrations and ArcA phosphorylation.

Aerobic conditions				
Strains	UQ(nmoles/g)	DMK(nmoles/g)	MK(nmoles/g)	ArcA – P(%)
**WT**	1146±330	86±35	82±32	6.0±3.7
***ΔubiCA***	0	346±92	427±226	7.0±4.4
***ΔmenA***	993±495	0	0	10.2±6.3
***ΔubiE***	0	321±42	0	4.2±3.3
**Anaerobic conditions**				
**Strains**	**UQ** **(nmoles/g)**	**DMK** **(nmoles/g)**	**MK** **(nmoles/g)**	**ArcA – P** **(%)**
**WT**	546±57	138±11	414±26	54.6±0.3
***ΔubiCA***	0	404±79	922±282	26.9±1.3
***ΔmenA***	644±195	0	0	62.7±0.3
***ΔubiE***	0	749±140	0	36.1±7.0
**Anaerobic+fumarate conditions**				
**Strains**	**UQ** **(nmoles/g)**	**DMK** **(nmoles/g)**	**MK** **(nmoles/g)**	**ArcA – P** **(%)**
**WT**	215±7	91±9	234±13	33.5±11.6
***ΔubiCA***	0	58±2	204±121	17.7±9.4
***ΔmenA***	391±178	0	0	31.4±14.2
***ΔubiE***	0	334±175	0	26.2±7.7

Total ubiquinone content (nmoles/g), demethylmenaquinone content (nmoles/g), menaquinone content (nmoles/g) and ArcA phosphorylation (%) for MG1655 (Wild type) and its quinone mutants during exponential growth in Evan’s medium supplied with 50 mM glucose and 1% LB at 37°C under aerobic, anaerobic and anaerobic with 50 mM fumarate conditions. The amount of quinone (nmoles/g) is expressed in nanomoles per gram dry cell weight. The values represent the mean of measured values from biological triplicates with standard deviation. WT: K12-Wild type.

### Relationship between Quinones and ArcAB Phosphorylation

In previous reports from our group [24], [29] and others [31], the relative level of ArcA phosphorylation was measured indirectly via a LacZ-based reporter enzyme assay. With this method the relative ArcA-P level is measured in the *E. coli* MC4100 derivative ASA12 via binding of ArcA-P to a modified *cydAB* promoter, which controls expression of the reading frame of *lacZ,* and was engineered to be ArcA-P selective [24]. The method to measure the level of ArcA phosphorylation used here, *i.e.* via Phos-tag SDS-PAGE, however, is a more direct method to measure this parameter (fig. S1) [1]. We observed that in all mutants grown aerobically, the level of ArcA phosphorylation is decreased compared to cells grown anaerobically. Based on the low phosphorylation level of ArcA in AV36 (*ΔubiE*) under aerobic conditions, we conclude that demethylmenaquinone is also able to inhibit kinase activity of ArcB, just as ubiquinone does (Table 3).

ArcA phosphorylation in the three batch conditions selected (i.e. aerobically, anaerobically and anaerobically plus fumarate) clearly increases from aerobic to anaerobic conditions. Furthermore, a small increase in ArcA phosphorylation was observed in anaerobic conditions when the external electron accepter is omitted, irrespective of the type of quinone present (Table 3). Therefore, the relative level of ArcA phosphorylation obtained anaerobically in the presence of fumarate is intermediate between the two extremes of aerobic- and anaerobic cells. This data clearly reflects previous results by Georgellis and coworkers, who first showed the role of UQ_0_ in ArcB kinase activity inhibition [21]. However, in addition to this, the observations in AV33 (*ΔubiCA*) and AV36 (*ΔubiE*) clearly show a role for napthaquinones in the regulation of ArcAB phosphorylation, such that the (oxidized) quinones (i.e. DMK and MK) decrease the level of ArcA phosphorylation. Furthermore, under anaerobic conditions the level of ArcA phosphorylation is consistently higher in AV36 (*ΔubiE*) than in AV33 (*ΔubiCA*), which indicates (D)MK to be sufficient for ArcB kinase inactivation under oxidizing conditions (Table 3). Based on this data the role of MK cannot be deciphered in detail because a strain containing MK only is not available. In future work it may be worthwhile to overexpress *ubiE*, to find out to what extent this may reduce DMK levels.

Considering the way UQ_0_ inhibits ArcB activity *in vitro*, the data on the relative level of ArcA-P under aerobic conditions suggests that ArcA phosphorylation is inhibited by increasing concentrations of (oxidized) ubiquinone. Furthermore, the data from anaerobic conditions supports this, as the oxidized/reduced ratio of the ubiquinones in AV34 (*ΔmenA*) is likely much lower than that of the menaquinones in AV33 (*ΔubiCA*). This assumption is based on the more negative redox midpoint potential of the demethylmenaquinone and menaquinone. Strikingly, under anaerobic conditions, the total concentration of ubiquinone is higher in AV34 (*ΔmenA*) than in the wild type. This is possibly caused by up-regulation of ubiquinone biosynthetic enzyme(s), or their activity, in AV34 (*ΔmenA*), so as to compensate for the absence of menaquinones. Additionally, the fact that the level of ArcA phosphorylation is slightly higher in AV34 (*ΔmenA*) than in the wild type (MG1655), may be due to the absence of (oxidized) menaquinones in this strain.

**Figure 1:**
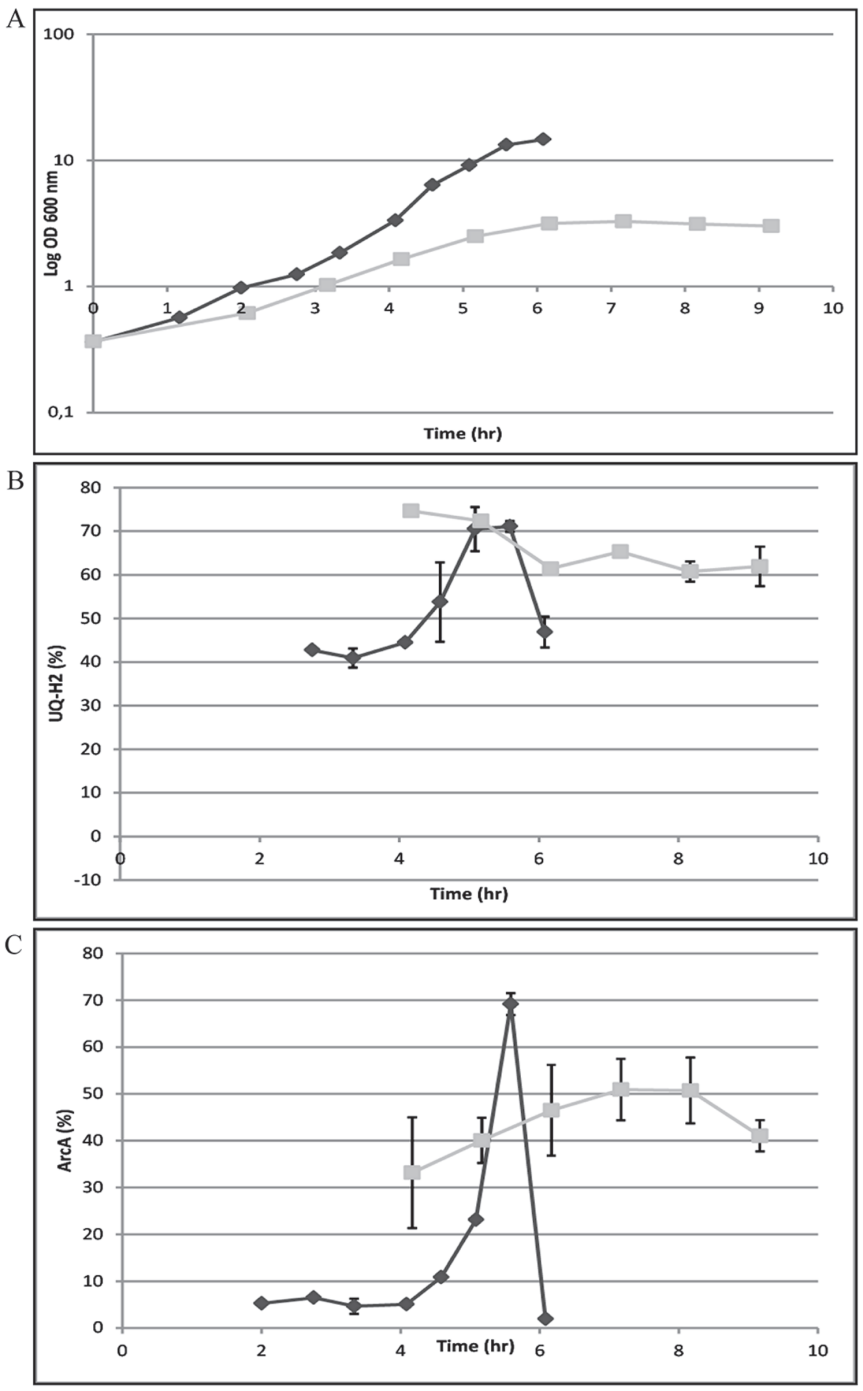
Growth phase dependence of relative ubiquinol content (%) and ArcA-P activity (%) for MG1655 (Wild type) aerobic and anaerobic batch conditions. A) Growth curve for MG1655 on Evan’s medium supplied with 20 mM glucose under batch conditions at 37°C. Light line; trend for OD_600_ under anaerobic conditions, Dark line; trend for OD_600_ under aerobic conditions. The data is from a single representative experiment and error bars are indicated for each value based on technical triplicates. B) Relative ubiquinol content (%) for MG1655 on Evan’s medium supplied with 20 mM glucose under batch conditions at 37°C. Light line; trend for ubiquinol content (%) under anaerobic conditions, Dark line; trend for ubiquinol content (%) under aerobic conditions. The data is from a single representative experiment and error bars are indicated for each value based on technical triplicates. C) ArcA-P (% (of total ArcA content)) for MG1655 grown on Evan’s medium supplied with 20 mM glucose under batch conditions at 37°C. Light line; trend for ArcA-P (%) under anaerobic conditions, Dark line; trend for ArcA-P (%) under aerobic conditions. The data is from a single representative experiment and error bars are indicated for each value based on technical triplicates.

In order to investigate the role of the napthaquinones in more detail and to further verify the above observations, it is essential to be able to differentiate between the reduced and oxidised forms of MK and DMK. So far, however, this latter issue has remained unsolved, due to a very high auto-oxidation rate of napthaquinones during their isolation and subsequent HPLC analysis. The total DMK and MK content are shown in Table S2. As an alternative we analysed the trend between growth (Fig. 1A), the relative amount of reduced ubiquinone (Fig. 1B) and the percentage of ArcA phosphorylation (Fig. 1C) in the wild type strain, as a function of time, in a batch culture grown aerobically and anaerobically.

Total quinone content (expressed as nmol/g dry weight) remains rather constant during all phases of growth in these batch cultures, which is according to expectations. The relative level of UQ_8_H_2_ (or: the UQ_8_H_2_/UQ_8_ ratio), however, increased rapidly during exponential growth, due to the limited availability of dissolved oxygen at increasing cell densities [29]. Upon entry into the stationary phase, the relative UQ_8_H_2_ content started to decrease as a response to restoration of complete oxygen saturation (Fig. 1B) due to a lack of electron donors in the cells. Interestingly, the trend of ArcA phosphorylation (here measured with the ‘phos-tag approach’; see Materials and Methods) slowly follows the relative level in reduction of ubiquinone (Fig. 1C). This correlation is restored at the end of the logarithmic phase, in which the level of ubiquinone is low (higher percentage of UQH_2_), and therefore the level of ArcA-P is high (Fig. 1A, B and C). Under anaerobic conditions, in contrast, the ArcA-P level is high from the early phases of exponential growth onwards, just as the relative level of UQ_8_H_2_.

This difference between the aerobic- and anaerobic batch experiment is fully consistent with a role of both the ubiquinones and the napthaquinones in the regulation of ArcB activity. The napthaquinones presumably stay longer in an oxidized state when, due to limited oxygen inflow, the ubiquinones are converted to their ubiquinol form (e.g. between 2 and 4 hrs in Fig. 1B), thus keeping ArcB activity and ArcA phosphorylation low. As the cells approach stationary phase, when oxygen is almost fully depleted (data not shown), the total quinone pool becomes reduced, which ends inhibition of ArcB kinase activity. Whether or not MK and DMK are more or less reactive towards ArcB than UQ can only be resolved once it is possible to measure the separate redox forms of one or both of these napthaquinones.

## Conclusion

A complex, indirect relationship exists between oxygen availability and ArcBA activity, which is caused by the sensitivity of ArcB activity to the redox state of its key cysteine residues. This generates a dependence of ArcB activity on the redox state of cellular redox-active metabolites. Previous results [24] have indicated that the redox-sensitivity of ArcB is related to the level of phosphorylation of ArcA and the presence of quinones in the cytoplasmic membrane. The experiments reported here unequivocally show an inhibitory role of both ubiquinone and napthaquinones(s) on ArcB kinase activity *in vivo*.

### Note Added in Preparation

While this manuscript was in preparation, Alvarez *et al*. (2013) [32] published a study that also shows that (D)MK can inactivate ArcB. Surprisingly, these authors also report that UQH_2_ cannot activate ArcB, which is in contrast to the results presented here and their own previous work (Georgellis *et al*. (2001) Science 292, 2314) [21].

## Supporting Information

Figure S1 **Representative picture of a Phos-tag gel.** The upper band represents the phosphorylated form of ArcA (corresponding to 35 kD) and the lower band represents the un-phosphorylated form of ArcA (corresponding to 28 kD). This gel shows samples from exponential-phase cultures grown under anaerobic batch conditions in Evan’s medium supplemented with 50 mM glucose and 1% (v/v) LB at 37°C. The lanes from left to right are loaded with A: *ΔubiE*, B: *ΔmenA*, C: *ΔubiCA*, D: MG1655 (wild type) and E: molecular weight marker.
(TIF)

Table S1 **Calculations and comparison of total intensities of ArcA and ArcA-P per sample.**
(DOC)

Table S2 **Total DMK and MK content for MG1655 (wild type) under aerobic and anaerobic conditions.**
(DOCX)
