# Supplementary figures and images for: Kinase Activity of ArcB from Escherichia coli Is Subject to Regulation by Both Ubiquinone and Demethylmenaquinone
Source: PLoS One. 2013 Oct 7;8(10):e75412. doi: 10.1371/journal.pone.0075412 (PMC3792059; doi:10.1371/journal.pone.0075412)

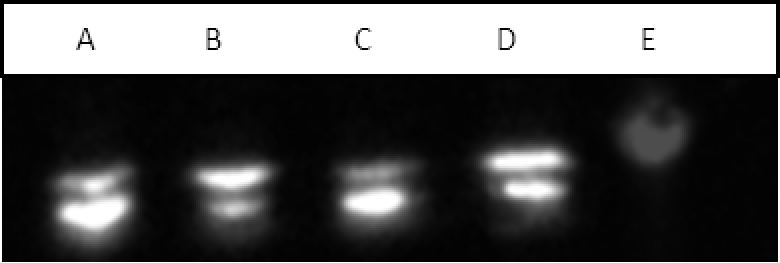

Supplement: Figure S1 — Representative picture of a Phos-tag gel. The upper band represents the phosphorylated form of ArcA (corresponding to 35 kD) and the lower band represents the un-phosphorylated form of ArcA (corresponding to 28 kD). This gel shows samples from exponential-phase cultures grown under anaerobic batch conditions in Evan’s medium supplemented with 50 mM glucose and 1% (v/v) LB at 37°C. The lanes from left to right are loaded with A: ΔubiE, B: ΔmenA, C: ΔubiCA, D: MG1655 (wild type) and E: molecular weight marker. (TIF) [file pone.0075412.s001.tif]
