# Supplementary material for: Kinase Activity of ArcB from Escherichia coli Is Subject to Regulation by Both Ubiquinone and Demethylmenaquinone
Source: PLoS One. 2013 Oct 7;8(10):e75412. doi: 10.1371/journal.pone.0075412 (PMC3792059; doi:10.1371/journal.pone.0075412)
Supplement: Table S1 — Calculations and comparison of total intensities of ArcA and ArcA-P per sample. (DOC) [file pone.0075412.s002.doc]

**Table S1 Calculations and comparison of total intensities of ArcA and ArcA-P per sample**

It is representative for all the other calculations. The data represents the cultures grown under batch anaerobic conditions in Evan’s medium supplemented with 50 mM glucose and 1% LB at 37^o^C in exponential phase. The unit of signal is arbitrary, based on intensity, as defined in software Image studio.

| **Strains in anaerobic cultures** | **ArcA phosphorylation %** | **Unphosphorylated ArcA band intensity (Signal)** | **Phosphorylated ArcA band intensity (Signal)** | **Total ArcA band intensity (Signal)** |
| --- | --- | --- | --- | --- |
| **Wild type** | 54,6 ± 0,3 | 0.378 ± 0.110 | 0.456 ± 0.138 | 0.833 ± 0.247 |
| ***ΔubiCA*** | 26,9 ± 1,3 | 0.556 ± 0.145 | 0.201 ± 0.040 | 0.757 ± 0.185 |
| ***ΔmenA*** | 62,7 ± 0,3 | 0.272 ± 0.063 | 0.456 ± 0.100 | 0.728 ± 0.163 |
| ***ΔubiE*** | 36,1 ± 7,0 | 0.619 ± 0.270 | 0.315 ± 0.051 | 0.933 ± 0.320 |
