# Supplementary material for: Kinase Activity of ArcB from Escherichia coli Is Subject to Regulation by Both Ubiquinone and Demethylmenaquinone
Source: PLoS One. 2013 Oct 7;8(10):e75412. doi: 10.1371/journal.pone.0075412 (PMC3792059; doi:10.1371/journal.pone.0075412)
Supplement: Table S2 — Total DMK and MK content for MG1655 (wild type) under aerobic and anaerobic conditions. (DOCX) [file pone.0075412.s003.docx]

**Table S2 Total DMK and MK content for MG1655 (wild type) under aerobic and anaerobic conditions**

The data represent the total DMK and MK content in MG1655 under aerobic (Left) and anaerobic (Right) batch growth conditions in Evan’s medium supplemented with 50 mM glucose and 1% LB at 37^o^C for the single representative experiments corresponding to Figure 1A, B and C.

| **Time (hr)** | **Total DMK (nmoles/gm)** | **Total MK (nmoles/gm)** |
| --- | --- | --- |
| **0.0** | N.D. | N.D. |
| **1.2** | N.D. | N.D. |
| **2.0** | N.D. | N.D. |
| **2.8** | N.D. | N.D. |
| **3.3** | N.D. | N.D. |
| **4.1** | N.D. | N.D. |
| **4.6** | N.D. | N.D. |
| **5.1** | 135.3 ± 51.0 | 7.4 ± 3.4 |
| **5.6** | 259.1 ± 2.5 | 30.0 ± 6.4 |
| **6.1** | 1.6 ± 0.4 | 74.6 ± 5.4 |

| **Time (hr)** | **Total DMK (nmoles/gm)** | **Total MK (nmoles/gm)** |
| --- | --- | --- |
| **0.0** | N.D. | N.D. |
| **2.1** | N.D. | N.D. |
| **3.2** | N.D. | N.D. |
| **4.2** | 499.8 ± 0.0 | 396.0 ± 0.0 |
| **5.2** | 454.4 ± 0.0 | 429.3 ± 0.0 |
| **6.2** | 393.2 ± 0.0 | 505.0 ± 0.0 |
| **7.2** | 349.8 ± 54.0 | 420.0 ± 8.5 |
| **8.2** | 461.5 ± 11.7 | 560.2 ± 41.2 |
| **9.2** | 392.7 ± 0.0 | 544.1 ± 0.0 |
